# Production of Volatile Compounds Using Wild Yeasts in a Cocoa Leachate-Based Culture Medium

**DOI:** 10.1021/acsomega.5c08827

**Published:** 2025-12-23

**Authors:** Claudia Johanna Sandoval-Lozano, Yanine Yubisay Trujillo Navarro, Luis Javier Lopez-Giraldo

**Affiliations:** † Grupo de Investigación en Ciencia y Tecnología de Alimentos, Escuela de Ingeniería Química, 28014 Universidad Industrial de Santander, 27th Avenue, ninth Street, Bucaramanga 680006, Santander, Colombia; ‡ Grupo de Investigación en Ingeniería y Tecnología de Alimentos, Facultad de Ingenierías y arquitectura, 27995 Universidad de Pamplona, Km 1 University City, Pamplona 543057, Norte de Santander, Colombia

## Abstract

Volatile organic compounds (VOCs) play a crucial role in cocoa fermentation, shaping its final aromatic profile. The aim of this research was to identify the volatile compounds produced by wild yeasts grown in a culture medium derived from cocoa leachate. VOCs were analyzed by headspace solid-phase microextraction coupled with gas chromatography–mass spectrometry and odor activity value screening. A total of twenty-six VOCs produced by yeast strains were identified and classified into six families: alcohols, esters, aldehydes, acids, ketones, and pyrazines. Ethanol was the most abundant (355.28 mg/kg), while the total ester concentration reached 105.84 mg/kg. Principal component analysis revealed the separation of the yeast into three main clusters. Strain Y33 belonged to the first cluster, which was the highest producer of ethanol and esters associated with fruity perceptions. In the second cluster, Y29a and Y244 were the main producers of alcohols related to floral perceptions. In the third cluster, ten yeasts were grouped by their ability to produce esters. Among them, Y195 and Y110MRS stood out for producing ethyl octanoate and 3-methybutanal, compounds associated with fruit and chocolate perceptions. Overall, our findings demonstrate that cocoa leachate, the main carbon source under real fermentation conditions, can generate a VOC profile linked to desirable sensory attributes and thus represents an excellent medium for VOC production with wild yeasts while contributing to the valorization of an agro-industrial byproduct.

## Graphical abstract

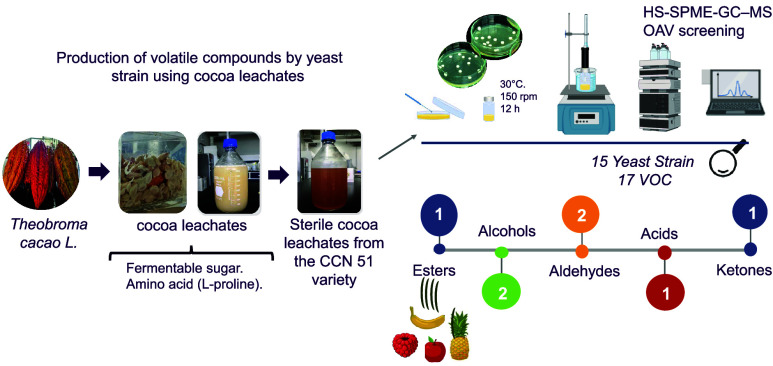

## Introduction

Volatile organic compounds (VOCs) are low-molecular-weight molecules (<300 Da) characterized by high volatility, low polarity, and low water solubility. These compounds are end-products or intermediates of microbial metabolic activity and play a key role in the formation of the aroma and flavor profiles of fermented food products such as wine, beer, coffee, and cocoa.^1^ During fermentation, yeasts metabolize sugars, yielding ethanol, CO_2_, and secondary metabolites, including alcohols, aldehydes, esters, organic acids, ketones, and terpenes. These metabolites are major contributors to the sensory characteristics of the final products.^2–8^

Several analytical techniques are available for the extraction and identification of volatile compounds from food matrices; however, the most widely employed is Headspace Solid-Phase Microextraction coupled with Gas Chromatography–Mass Spectrometry (HS-SPME-GC-MS), which enables sensitive and selective quantification of VOCs. High-molecular-weight flavor compounds containing more than ten carbon atoms can be efficiently extracted and detected using this technique. This method provides excellent precision, high sensitivity, and favorable operational conditions. Additionally, it can be combined with the Odor Activity Value (OAV) to relate VOC concentration to their odor threshold value (OTV), providing insights into the sensory relevance of each compound.^9^ In a previous study, we developed a methodology using a synthetic culture medium to select wild yeasts isolated from cocoa bean fermentations based on their potential to produce volatile compounds associated with desirable flavor attributes. Thirty-six VOCs were identified and classified into six families: alcohols, esters, aldehydes, acids, ketones, and pyrazines. The yeast strains were grouped according to their floral or fruity aromatic profiles.^10^ However, that study did not consider the influence of the culture growth medium, an important factor in on-farm wooden-box fermentations, where the carbon source is a mixture of complex sugars rather than the single glucose composition of synthetic media.

The availability of nutrients in the fermentation medium, particularly the carbon source, strongly influences VOC production. Medium complexity affects yeast metabolism, physiology, and interactions.^11,12^ Consequently, variations in substrate composition can modify the concentration of volatile compounds, directly affecting the fermentation performance and determining the sensory quality of the final products.

Synthetic culture media are chemically defined systems that contain specific concentrations of carbon and nitrogen sources. They are easy to prepare and provide reproducible conditions for microbial growth, including yeast cultivation.^12^ Among these, Sabouraud Dextrose Broth (SDB) contains 20 g/L glucose as the primary carbon and energy source. It is formulated with peptones that supply amino acids that are essential for yeast metabolism. The combination of glucose as the main carbon source and the acidic nature of the medium influences yeast metabolic pathways, promoting alcoholic fermentation and enhancing the biosynthesis of aroma-related metabolites.^10^

Conversely, natural culture media derived from agro-industrial byproducts, such as cocoa leachate, offer a sustainable alternative that increases nutrient diversity while reducing food waste. Cocoa leachate represents approximately 5–10% of the fresh weight of cocoa pulp.^13,14^ On a typical two-ha plantation in Santander, Colombia, producing approximately six tons of cocoa, nearly 600 kg of mucilage, equivalent to 500–700 L of cocoa leachate, could be used for yeast production.

Cocoa leachate is a whitish, milky white liquid extracted from the mucilaginous pulp surrounding cocoa beans. It is an agro-industrial byproduct resulting from pulp liquefaction during cocoa fermentation, produced by enzymatic degradation of pectin. It is rich in fermentable sugars, pectins, proteins, and trace elements, which confer high viscosity and favorable physicochemical and enzymatic properties, making it a suitable carbon source for microbial fermentation in various biotechnological applications.^13–15^

Previous studies have evaluated yeast strain selection for fermentative processes, highlighting the complexity and variability of VOC production that arise from strain-dependent metabolic traits and differences in carbon source availability.^2,4,5^ However, no studies to date have examined the influence of using cocoa mucilage as the primary carbon source on the VOC profiles produced by wild yeasts. This information is essential for guiding the selection of starter cultures and for reducing uncertainties associated with the use of conventional synthetic media. Furthermore, validating cocoa leachate as a viable fermentation substrate would facilitate technology transfer to cocoa farms, where this resource is abundant yet largely underused. Its improper disposal into soils and waterways has also become an environmental concern. The use of natural culture media could, therefore, contribute significantly to the development of starter cultures that enhance the sensory quality of cocoa and its derived products. Understanding and optimizing the interactions among yeast metabolism, culture media, and volatile compound formation are crucial to achieving this objective.

The aim of the present study was to characterize the volatile compound profiles produced by wild yeasts grown in a culture medium derived from cocoa leachate and to compare them with those produced in a medium containing glucose as the sole carbon source. This approach is essential for selecting the most suitable yeast consortia for box fermentations in which cocoa leachate serves as the carbon source, therefore reducing uncertainties associated with metabolic shifts that occur when glucose is used instead of the natural mixture of sugars present in cocoa leachate.

## Materials and Methods

### Yeast Strains

In this study, 17 yeast strains previously isolated from the spontaneous fermentation of cocoa beans and preserved at −80 °C in 20% (w/v) glycerol in the culture collection established by the Grupo de Ciencia y Tecnología de Alimentos (CICTA) at the Universidad Industrial de Santander (UIS), Colombia, were used. The database of this collection is accessible through the Colombian Biodiversity Information System (10.15472/n6uwoc). Strains Y29a, Y33, Y85, and Y110mrs were registered under the contract on access to genetic resources and derived products from Colombia (registration number 303).

### Reactivation of Yeasts

The yeast strains were reactivated and grown on Sabouraud Dextrose Agar (SDA) plates (Merck, Darmstadt, Germany), supplemented with 100 mg/L chloramphenicol (Sigma-Aldrich, Steinheim, Germany), and incubated at 30 °C for 5 days.

### Natural Culture Medium

The production of volatile compounds was evaluated by using a natural medium consisting of cocoa leachates obtained from cocoa beans of the CCN 51 variety (Castro Naranjal Collection 51). The medium was prepared as follows: cocoa fruits were opened, and the fresh beans were manually extracted and then placed in perforated boxes to allow the liquid to be collected by filtration. The collected liquid was transferred to airtight containers and sterilized in an autoclave at 121 °C for 15 min. It was subsequently stored at 4 °C for future use. The natural medium was adjusted to a fermentable sugar concentration of 20 g/L and supplemented with 1% peptone as an external nitrogen source. This supplementation was performed to reduce the cultivation time required to reach the stationary phase.^16^

The fermentable sugar content was determined using a High-Performance Liquid Chromatograph (HPLC) system (Thermo Dionex Ultimate 3000) equipped with a degasser, an automatic injector, a refractive index detector, and a COREGEL 107H (7.8 mm × 300 mm, 8 μm) column maintained at a constant temperature of 30 °C. The mobile phase consisted of water acidified with 8 mM H_2_SO_4_ in isocratic mode, with a 20 μL injection volume and a 30 min run time per sample.^16^

The protein content was determined using the volumetric Kjeldahl method according to CICTA Internal Method GOMEPL.01, using a conversion factor of 6.25 (ISO 1871:2009). Nitrogen content in the form of amino acids was determined following the procedure proposed by Balladares et al.^13^ For this, the cocoa leachate was freeze-dried using a LABCONCO unit at a vacuum pressure of 0.09 mbar and −80 °C. Subsequently, 2 mg of freeze-dried sample was derivatized with 200 μL of *N*,*O*-bis­(trimethylsilyl)­trifluoroacetamide (BSTFA) (Sigma-Aldrich). The supernatant was analyzed by gas chromatography coupled to mass spectrometry (GC-MS) on a 7890A gas chromatograph equipped with a 5975C triple quadrupole mass selective detector (Agilent Technologies). An HP-5 MS capillary column (30 m × 0.25 mm × 0.5 μm) was used as the stationary phase. The oven temperature ramp was as follows: 80 °C for 1 min, then increased at 7 °C/min until it reached 300 °C. Helium was used as the carrier gas with a constant flow velocity of 1.5 mL/min. Finally, the compounds were identified by comparing the experimental mass spectra with those reported in the Wiley and NIST 2011 mass spectral libraries.

### Volatile Compound Analysis

The volatile compounds produced by yeasts were determined by HS-SPME-GC–MS according to Sandoval-Lozano et al., with slight modifications.^10^

### Sample Preparation

Each yeast strain was grown on SDA at 30 °C for 48 h. Subsequently, a single colony was inoculated into 3 mL of the fermentation medium in sterile vials of 10 mL sealed with PTFE/silicone septa and fitted with a magnetic stirrer of 4 mm × 12 mm. The vials were incubated at 30 °C and 150 rpm for 12 h in a Being Shaker (Quimicompany). At the end of the incubation period, 1 mL of the culture was withdrawn using a hypodermic needle to quantify Colony Forming Units (CFU) by serial dilution and plating on SDA agar. Under the same conditions, control vials containing only 3 mL of medium were evaluated to identify the volatile compounds present in the cocoa leachate.

### Extraction of Volatile Compounds

Extraction was performed by adsorption. The vial was placed in a mineral oil bath at 60 °C for 15 min to allow the volatile fraction to transition from the liquid phase to the headspace of the vial. Then, the extraction fiber was inserted into the headspace for 40 min at 60 °C under constant agitation. A 50/30 μm thick DVB/CAR/PDMS fiber (Supelco, Bellefonte, PA) was used.

### Gas Chromatography

At the end of the extraction, the SPME fiber was desorbed in the injection port of a Hewlett-Packard 7890A gas chromatograph coupled to an HP 5972 mass selective detector (Agilent Technologies) at 265 °C, in split mode, for 7 min. Compound separation was carried out on an HP-5 column (30 m × 0.25 mm × 0.25 μm). The oven temperature program was as follows: 30 °C for 10 min, increasing at 3 °C/min to 60 °C, then at 10 °C/min up to 150 °C, and finally 4 °C/min up to 200 °C. Nitrogen was used as the carrier gas at a constant flow rate of 1 mL/min. Electron impact ionization was set at 70 eV, and the ion source temperature was maintained at 230 °C.^10^

### Quantification of Volatile Compounds

Quantification was carried out using toluene as an internal standard at a concentration of 4200 mg/L in methanol. To calculate the concentration of each compound, the volatiles produced by the fermentation medium (control) were evaluated. The concentration of each compound was expressed as milligrams per kilogram and was calculated using eq 1

1 $$\mathrm{VOC}=\left(\mathrm{RF}\times {a\mathrm{rea}}_{\mathrm{voc}}\times \mathrm{IS}/{a\mathrm{rea}}_{\mathrm{IS}}\right)$$

where VOC is the volatile organic compound concentration, area_voc_ is the volatile organic compound area, Area_IS_ is the internal standard area, IS is the concentration of the internal standard, and RF is the response factor of the family.^17^

### Calculation of OAV

To estimate the contribution of volatile compounds produced by each yeast strain, concentration data were transformed into OAVs by dividing the observed volatile compound concentration by the odor threshold values (OTVs) obtained from the literature. This screening was conducted to determine which volatile compounds exceeded their threshold value (>1), to identify those with the highest potential impact on the aroma profile generated by each yeast strain.^10,18,19^

### Statistical Analysis

Each yeast strain was analyzed in triplicate, and the experimental results were expressed as the mean ± standard deviation (SD). To evaluate the contribution of yeast strains in terms of volatile component production, Principal Component Analysis (PCA) and Hierarchical Cluster Analysis (HCA) were performed using Unscrambler X software (version 10.5.1, *CAMO Inc., Oslo, Norway*). For these analyses, volatile compounds and yeast strains that did not present an OAV throughout the matrix were excluded. Then, volatile compound concentrations were standardized as *z*-scores. For HCA, Ward′s minimum variance method and half-squared Euclidean distances were used.

Differences in volatile compound concentrations produced by yeasts in a synthetic versus natural media were analyzed using the nonparametric Mann–Whitney *U* test. Data normality was checked by the Shapiro-Wilk test. These analyses were performed using Statistic software (version 14.0.015, TIBCO), with significance set at *p* < 0.05.

## Results and Discussion

### Cocoa Leachate

In the present study, sterile leachate from CCN 51 cocoa contained 4.87% glucose, 7.24% fructose, and 0.62% sucrose (Figure 1a,b). The principal sugars identified in this study were similar to those previously reported in cocoa pulp or leachate; however, their concentrations differed from the values found in the literature. For example, Saavedra-Sanabria et al. reported 8.69% glucose, 9.08% fructose, and 0.027% sucrose in sterile leachates from CCN 51 cocoa collected in San Vicente de Chucurí, Santander, Colombia.^15^ Similar results were obtained by Balladares et al., who reported 2.13% glucose, 4.42% fructose, and 2.15% sucrose in leachates from fresh cacao beans from Ecuador.^13^ Likewise, Quimbita et al. found 2.79% glucose, 3.06% fructose, and 6.11% sucrose in sterile leachates from CCN 51 cocoa from the same region.^20^ Interestingly, in most reports, fructose appears in the highest proportion, followed by glucose and then sucrose, except for the profile reported by Quimbita et al. These variations could be attributed to factors such as agroclimatic conditions, the cocoa genotypes used for leachate collection, and the ripeness stage of the cocoa pods. Despite the observed differences in sugar content, the presence of these fermentable sugars makes cocoa leachate an excellent carbon source for yeast growth, supporting its use as a tarter culture medium in fermentation processes.

**Figure 1:**
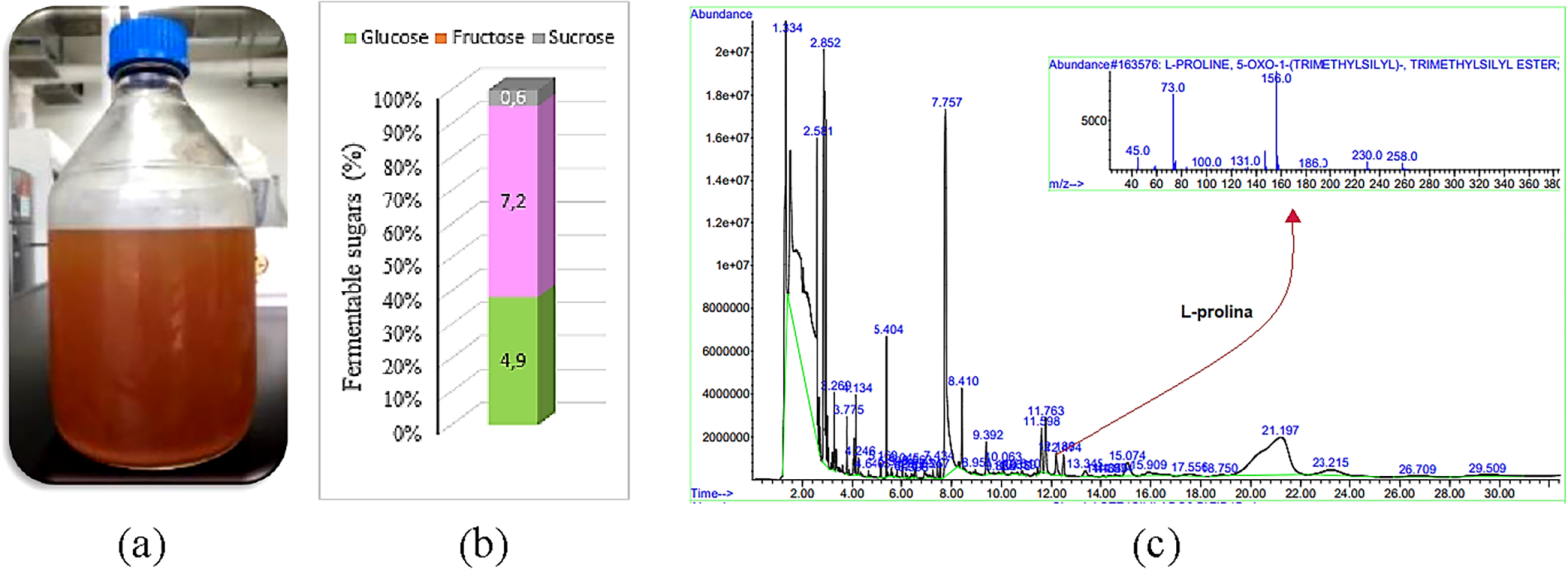
(a) Sterile cocoa leachate from the CCN 51 variety. (b) Fermentable sugar content, expressed as the mean ± standard deviation (SD) (*n* = 5). (c) Chromatographic profile and mass spectrum.

Another essential nutrient for yeast growth and metabolic activity is nitrogen. The CCN 51 cocoa leachate contained 0.05% ± 0.002 nitrogen by mass (Table 1), and the amino acid l-proline (Pro) was detected with a quality score of 86 and a retention time of 12.186 min (Figure 1c).

**Table 1:** Ammonia Nitrogen Content in Cocoa Leachate from CCN 51^a^

**Parameters**	**Batch**			
	1	2	3	mean
protein (%)	0.31	0.33	0.30	0.313 ± 0.001
nitrogen equivalent (% by mass)	0.049	0.052	0.048	0.050 ± 0.002

a Data are present as mean ± standard error (*n* = 3).

Hence, it is likely that nitrogen present in the cocoa leachate can be assimilated by yeast strains, becoming a key factor for their multiplication and physiological activity. Balladares et al. identified two amino acids, aspartic acid (Asp) and glutamic acid (Glu), in leachates from Ecuadorian cocoa.^13^ These amino acids can be used by yeast as a nitrogen source. Previous studies conducted by our research group demonstrated that cocoa leachate can be used as a culture medium for the growth of *Saccharomyces cerevisiae* without the need for an external nitrogen source. The yeast reached maximum biomass production (3.41 g/L) after 26 h, with a maximum productivity of 0.26 g/L.h achieved at 4 h.^16^ These results confirm that cocoa leachate contains available and assailable nitrogenous compounds, such as amino acids and peptides, that fulfill the nutritional requirements of yeast. According to Li et al.,the presence of amino acids in culture media promotes microbial biomass production by enabling yeast to adapt its metabolic pathways and reducing the need to synthesize amino acids.^21^

### Analysis of Volatile Compounds

Volatile compounds produced by 17 native yeast strains were identified using cocoa leachates from CCN 51 as the growth medium (Table 2). All strains reached cell densities of 1 × 10^8^ CFU/mL. A total of 26 VOCs were detected and grouped into six families: esters (*n* = 13), alcohols (*n* = 4), aldehydes (*n* = 3), acids (*n* = 2), ketones (*n* = 2), and pyrazines (*n* = 2). Ten compounds inherent to medium were identified: benzaldehyde (0.06 mg/kg), 3-methylbutanal (0.056 mg/kg), ethyl dodecanoate (0.166 mg/kg), ethyl 3-phenyl-2-propenoate (0.075 mg/kg), 3-propenyl 3-phenyl-2-propenoate (0.636 mg/kg), 2-phenylethyl acetate (0.439 mg/kg), methyl phenylacetate (0.060 mg/kg), phenylethyl ketone (0.004 mg/kg), 2,3,5,6-tetramethylpyrazine (0.039 mg/kg), and 2,3,5-trimethylpyrazine (0.034 mg/kg). The concentrations of these compounds were subtracted from those obtained from each yeast to eliminate the volatiles inherent to the cocoa leachate. Benzaldehyde was not produced by any of the yeast strains during fermentation, suggesting that it is an intrinsic compound of the culture medium rather than a yeast-derived metabolite. This finding is consistent with that of Koné et al., who identified benzaldehyde as a compound present in SDB and also as a metabolite produced by some yeasts.^22^ However, in this study, none of the strains produced benzaldehyde, which may indicate that it was metabolized by the yeasts. Previous research has shown that various yeast species are capable of synthesizing benzaldehyde.^23,24^ Therefore, it is possible that the yeasts analyzed in this research metabolized them during growth.

**Table 2:** Aromatic Profile of Native Yeasts Detected by SPME-GC-MS Grown in Cocoa Leachate from CCN 51^a^ ^,^ ^b^

**Volatile compounds**	**OTV (mg/kg)**	**NCM**	**Yeasts strains**																
			Y1	Y4	Y12	Y13a	Y17	Y19	Y29a	Y33	Y85	Y97	Y110 mrs	Y133	Y195	Y200	Y218	Y23	Y244
decanoic acid	1.1	n.d	0.19 ± 0.007	n.d	n.d	n.d	n.d	0.04 ± 0.009	0.52 ± 0.020	1.17 ± 0.450	0.34 ± 0.069	0.02 ± 0.004	0.01 ± 0.004	0.26 ± 0.102	n.d	n.d	n.d	0.03 ± 0.005	n.d
2-methylpropanoic acid	2.3	n.d	0.67 ± 0.001	0.02 ± 0.017	0.05 ± 0.012	0.08 ± 0.007	n.d	0.50 ± 0.102	1.55 ± 0.148	n.d	1.73 ± 0.295	n.d	0.12 ± 0.018	0.44 ± 0.267	n.d	0.06 ± 0.001	n.d	0.93 ± 0.096	2.03 ± 2.358
α-terpineol	0.33	n.d	n.d	n.d	n.d	n.d	n.d	n.d	n.d	n.d	n.d	n.d	0.04 ± 0.007	n.d	n.d	n.d	n.d	n.d	n.d
2-pentanol	4	n.d	n.d	n.d	n.d	n.d	n.d	0.39 ± 0.051	1.37 ± 0.148	n.d	1.20 ± 0.178	n.d	0.17 ± 0.012	n.d	0.35 ± 0.090	n.d	n.d	n.d	n.d
ethanol	30	n.d	28.6 ± 0.001	1.38 ± 0.018	8.25 ± 7.859	6.47 ± 2.524	3.11 ± 1.736	11.7 ± 9.916	45.3 ± 3.367	55.9 ± 6.709	22.2 ± 5.229	7.42 ± 4.553	6.34 ± 0.506	27.6 ± 8.238	12.8 ± 5.508	4.29 ± 0.001	50.4 ± 5.682	9.39 ± 1.681	59.7 ± 30.85
3-methyl-2-butanol	3	n.d	4.86 ± 0.005	0.21 ± 0.009	0.36 ± 0.082	0.59 ± 0.107	n.d	n.d	n.d	4.89 ± 4.311	n.d	n.d	0.04 ± 0.006	3.79 ± 1.172	n.d	0.52 ± 0.005	8.47 ± 0.521	n.d	8.28 ± 1.007
4-methyl-2-phenyl-2-pentenal	-	n.d	n.d	n.d	n.d	n.d	n.d	0.25 ± 0.104	0.80 ± 0.611	n.d	0.35 ± 0.106	n.d	0.08 ± 0.019	0.32 ± 0.295	0.48 ± 0.205	n.d	n.d	0.30 ± 0.377	1.23 ± 0.238
2-phenyl-2-butenal	1.7	n.d	0.21 ± 0.001	n.d	n.d	n.d	n.d	n.d	0.28 ± 0.121	n.d	0.27 ± 0.136	0.03 ± 0.010	0.09 ± 0.024	0.20 ± 0.071	n.d	n.d	n.d	n.d	0.86 ± 0.274
benzaldehyde	0.06	0.07 ± 0.000	n.d	n.d	n.d	n.d	n.d	n.d	n.d	n.d	n.d	n.d	n.d	n.d	n.d	n.d	n.d	n.d	n.d
3-methylbutanal	0.013	0.06 ± 0.042	n.d	n.d	n.d	n.d	n.d	n.d	n.d	n.d	n.d	n.d	4.52 ± 0.756	n.d	0.06 ± 0.018	n.d	n.d	n.d	n.d
ethyl dodecanoate	1.5	0.17 ± 0.055	2.10 ± 0.001	n.d	n.d	n.d	n.d	0.39 ± 0.043	1.39 ± 1.081	3.69 ± 1.843	0.65 ± 0.321	n.d	n.d	0.33 ± 0.304	0.86 ± 0.761	n.d	n.d	0.49 ± 0.038	2.41 ± 0.206
ethyl 3-phenyl-2-propenoate	0.002	0.08 ± 0.027	0.57 ± 0.001	n.d	n.d	n.d	n.d	0.05 ± 0.007	n.d	n.d	n.d	n.d	n.d	n.d	n.d	n.d	4.55 ± 2.248	0.08 ± 0.010	n.d
2-methylpropyl benzoate	-	n.d	2.57 ± 0.001	0.11 ± 0.004	n.d	n.d	n.d	n.d	2.33 ± 0.694	5.61 ± 0.252	1.13 ± 0.193	0.06 ± 0.022	n.d	n.d	n.d	n.d	n.d	n.d	n.d
3-propenyl 3-phenyl-2-propenoate	-	0.64 ± 0.114	2.04 ± 0.001	n.d	n.d	n.d	n.d	n.d	n.d	3.18 ± 1.523	0.009 ± 0.00	n.d	n.d	0.73 ± 0.428	0.90 ± 0.546	n.d	4.52 ± 4.340	n.d	1.55 ± 0.282
ethyl 3-phenyl propionate	0,005	n.d	n.d	n.d	0.14 ± 0.027	0.14 ± 0.023	n.d	n.d	n.d	n.d	n.d	n.d	n.d	n.d	n.d	0.13 ± 0.005	7.65 ± 7.029	n.d	n.d
ethyl decanoate	0.2	n.d	n.d	n.d	0.08 ± 0.021	0.10 ± 0.010	n.d	0.29 ± 0.090	4.00 ± 0.010	n.d	0.70 ± 0.061	0.05 ± 0.021	n.d	0.47 ± 0.025	n.d	0.0 ±	3.56 ± 2.833	0.19 ± 0.113	n.d
2-phenylethyl acetate	0.23	0.44 ± 0.065	1.12 ± 0.001	n.d	n.d	n.d	n.d	0.18 ± 0.014	0.45 ± 0.345	1.61 ± 0.454	n.d	n.d	n.d	0.03 ± 0.028	0.23 ± 0.201	n.d	n.d	0.17 ± 0.030	n.d
ethyl octanoate	0.58	n.d	n.d	0.70 ± 0.111	n.d	n.d	n.d	n.d	n.d	n.d	n.d	n.d	23.4 ± 2.903	n.d	1.63 ± 0.426	0.03 ± 0.001	n.d	n.d	n.d
methyl phenylacetate	-	0.06 ± 0.010	1.07 ± 0.001	n.d	0.12 ± 0.060	n.d	n.d	0.57 ± 0.140	n.d	2.29 ± 0.542	0.96 ± 0.143	0.006 ± 0.00	n.d	0.93 ± 0.195	n.d	0.04 ± 0.001	5.53 ± 0.403	0.54 ± 0.078	1.47 ± 0.926
benzyl acetate	124	00	n.d	n.d	n.d	0.39 ± 0.102	n.d	0.63 ± 0.190	n.d	1.31 ± 0.481	n.d	n.d	n.d	n.d	n.d	n.d	n.d	0.63 ± 0.052	1.52 ± 0.578
3-methylbuthyl acetate	0.16	n.d	1.21 ± 0.001	n.d	n.d	n.d	0.24 ± 0.162	n.d	n.d	1.75 ± 0.369	n.d	n.d	n.d	n.d	0.23 ± 0.183	0.05 ± 0.001	n.d	0.23 ± 0.035	n.d
2-methylpropyl acetate	1854	n.d	n.d	0.78 ± 0.046	n.d	n.d	n.d	n.d	n.d	n.d	n.d	n.d	n.d	n.d	n.d	n.d	n.d	n.d	n.d
1-phenylethanone	5.6	n.d	n.d	n.d	n.d	n.d	n.d	n.d	n.d	5.84 ± 1.076	n.d	n.d	n.d	n.d	n.d	n.d	n.d	n.d	n.d.
2,3-butanedione	0.005	0.003 ± 0.0	0.20 ± 0.005	n.d	0.01 ± 0.004	n.d	n.d	0.01 ± 0.003	n.d	n.d	n.d	0.01 ± 0.004	n.d	n.d	n.d	0.03 ± 0.001	0.68 ± 0.040	0.05 ± 0.012	0.24 ± 0.020
2,3,5,6-tetramethylpyrazine	--	n.d	n.d	n.d	0.006 ± 0.00	0.01 ± 0.005	n.d	n.d	n.d	n.d	n.d	n.d	n.d	0.03 ± 0.010	n.d	n.d	n.d.	n.d	0.06 ± 0.023
2,3,5-trimethylpyrazine	1.8	0.04 ± 0.007	0.03 ± 0.001	. n.d	0.00 n.d0	n.d	n.d	n.d	0.03 ± 0.005	0.09 ± 0.025	0.02 ± 0.004	n.d	n.d	n.d	n.d	n.d	n.d	n.d	n.d

a NCM: Natural culture medium, without addition of yeast. n.d: not detected. Data are present as mean ± standard error (*n* = 3).

b Odor Activity Values: Sandoval-Lozano et al.,^10^ Escobar et al.,^25^ Wang et al.,^26^ and Akoa et al.^18^

Subsequently, an OAV screening was performed for each compound in each yeast strain. Compounds with OAV values below the threshold (OAV > 1) were excluded, as well as yeast strains that did not produce any compounds above this threshold, since these would have an insignificant contribution to the overall aroma profile (Table S1). The eliminated compounds were: 2-methylpropanoic acid, α-terpineol, 2-pentanol, 2-phenyl-2-butenal, benzaldehyde, benzyl acetate, 2-methylpropyl acetate, phenylethyl ketone, 2,3,5,6-tetramethylpyrazine, and 2,3,5-trimethylpyrazine. Yeast strains Y17 and Y97 were also excluded, although they have previously been reported as ester producers when cultivated in SDB.^10^

On the other hand, compounds such as α-terpineol, 2-pentanol, 2-phenyl-2-butenal, and 2,3,5-trimethylpyrazine did not reach the minimum concentrations required to be perceived olfactory (OAV < 1). In this study, the concentrations of 2-pentanol and α-terpineol did not exceed the olfactory threshold in the cocoa leachate medium, indicating a limited contribution to the aroma profile under these conditions. In contrast, when glucose was used as a carbon and energy source, different yeast strains produced these compounds at concentrations that exceeded the perception threshold.^10^ Since volatile compounds with an OAV greater than one contribute to the aroma profile in fermentation processes,^18,19^ these results suggest that the carbon source strongly influences volatile metabolite production by yeasts.

The pyrazines detected in this study were excluded during the OAV screening. It is noteworthy that 2,3,5-trimethylpyrazine and 2,3,5,6-tetramethylpyrazine were produced only in trace amounts, likely as byproducts of amino acid metabolism and interactions with other compounds in the cocoa leachate medium (Table 2). This process generally occurs under stress conditions, such as nutrient limitation or oxygen restriction, or when the yeast cells are exposed to high concentrations of amines or pyrazine precursors.

As a result of the OAV screening, 15 yeast strains produced a total of 17 volatile compounds, grouped as follows: alcohols (*n* = 2), esters (*n* = 11), aldehydes (*n* = 2), acids (*n* = 1), and ketones (*n* = 1). Alcohols and esters were the predominant families, accounting for 76.77 and 21% of the total volatile compound concentration, respectively, whereas the remaining families presented concentrations below 1.7%. Ethanol, representing 70.52% of the total concentration, reached its highest value (355.28 mg/kg). Its relatively high odor threshold of 30 mg/kg,^19^ combined with its high concentration, allowed this compound to be olfactory perceived. This result was expected, as ethanol is a primary metabolic product derived from sugar fermentation and is directly related to yeast growth in the cocoa leachate medium. 3-Methyl-2-butanol is another product of yeast metabolism, desirable for its slightly sweet and fruity notes. With an odor threshold of 3 mg/kg, this compound contributes to the overall aroma profile. The production of these alcohols is directly related to the metabolic pathways used by yeast to transform compounds present in the culture medium. For example, higher alcohols such as 3-methyl-2-butanol are produced through the Ehrlich pathway, which involves transamination, decarboxylation, and subsequent dehydrogenation of amino acids, or through the anabolic pathway, as byproducts of amino acid biosynthesis from pyruvate.^27,28^ In the cocoa fermentation process, ethanol is typically produced during the anaerobic phase. Since alcohols can be oxidized to acetic acid or converted into esters, a high alcohol content is considered beneficial for producing cocoa products with floral or sweet notes.^29^

The second most abundant group was the esters with a total concentration of 105.84 mg/kg. These were categorized into acetate esters (e.g., ethyl acetate, 2-phenylethyl acetate (2-PEA), and isoamyl acetate) and ethyl esters (e.g., ethyl dodecanoate, ethyl 3-phenyl-2-propenoate, 2-methylpropyl benzoate, 3-propenyl 3-phenyl-2-propenoate, ethyl 3-phenyl propionate, ethyl decanoate, ethyl octanoate, and methyl-2-phenylacetate). Among these volatiles, ethyl acetate, formed by esterification of acetic acid and ethanol, is known for imparting tropical fruity notes (such as pineapple, sweet, and fruit).^30^ Its synthesis depends on the ethanol concentration. Regarding 2-PEA, its production can be explained by the esterification of 2-phenylethanol (2-PE) with acetic acid as well as the transesterification of 2-PE with acetate esters. This compound is considered a key aroma marker, contributing floral and fruity notes, with an OTV of 0.23 mg/kg.^29,31^

Unlike other studies that have reported the production of both 2-PE and 2-PEA,^10^ in this study, 2-PE was not detected in the cocoa leachate medium. This suggests that the precursor was fully metabolized to produce 2-PEA through a transesterification reaction. Its formation involves the transfer of an acetyl group from Acetyl-CoA to the hydroxyl group of 2-phenylethanol, leading to the synthesis of 2-PEA. Another possible explanation for the absence of 2-PE is the limited availability of l-phenylalanine in the cocoa leachate. In this case, the Ehrlich pathway, which converts the aromatic amino acid l-phenylalanine to 2-PE, proceeds through three main enzymatic steps. First, l-phenylalanine is transaminated to phenylpyruvate by aminotransferases Aro8p and Aro9p. Then, phenylpyruvate is decarboxylated to phenylacetaldehyde by Aro 10p and the pyruvate decarboxylases Pdc1p, Pdc5p, and Pdc6p. Finally, phenylacetaldehyde is reduced to 2-PE by several alcohol dehydrogenases (Adh1p and Adh5p) and the formaldehyde dehydrogenase Sfa 1p.^32,33^

On the other hand, the production of isoamyl acetate depends on the esterification of 3-methylbutanol by yeasts. This compound contributes sweet and fruity notes, particularly banana,^34^ with an odor threshold value of 34.25 mg/kg, indicating a positive contribution to aroma. Ethyl octanoate reached the highest concentration (25.8 mg/kg) among esters and notably exceeded its odor threshold value; therefore, yeast strains that produce this compound could contribute desirable fruity notes in controlled fermentations.

The main aldehyde produced by the yeasts was 3-methylbutanal, considered a key aroma compound contributing to the aromatic properties and quality of cocoa beans due to its pleasant malt and chocolate notes. The formation of this aldehyde can be attributed to the Strecker degradation pathway of the precursor amino acid leucine during fermentation. Strecker aldehydes are generated from the reaction between a specific amino acid and a dicarbonyl compound and are widely recognized for their contribution to flavor development.^29,30^ 3-Methylbutanal has a low OTV of 0.001 mg/kg, making it easily perceptible. Another compound identified was diacetyl (2,3-butanedione), a secondary yeast metabolite that imparts a buttery aroma to cocoa beans. The production of 2,3-butanedione reported in this study is consistent with the data provided by Erazo Solorzano et al., who also reported the production of this compound during cacao fermentation.^31^

### Principal Component Analysis

Seventeen volatile compounds obtained through OAV screening were used as variables to carry out a PCA in order to visualize the relationships between the volatile compounds and yeast strains.

Two principal components were extracted, accounting for 63% of the total variance: PC1 (33%) and PC2 (27%) (Figure 2a). According to the biplot loadings, the yeasts showed associations with an aromatic profile dominated by esters. Strain Y33, located in the positive region PC1, showed strong correlations with decanoic acid (C1), ethyl dodecanoate (C6, fruity), 2-methylpropyl benzoate (C8, fruity), 2-phenylethyl acetate (C12, fruity and floral), and ethyl acetate (C15, fruity). Additionally, strain Y29a also appeared in the positive PC1 region and was correlated with 4-methyl-2-phenyl-2-pentenal (C4, floral, sweet). Ethanol (C2) and strain Y1 were also located within this same quadrant. In the positive region of PC2, compounds such as 3-methyl-2-butanol (C3, floral), ethyl 3-phenyl-2-propenoate (C7), and methyl-2-phenylacetate (C14, honey, rose, floral) were associated with strains Y218 and Y244, indicating that these yeasts contribute to floral notes. Strains Y110mrs and Y195 were linked to 3-methylbutanal (C5, chocolate) and ethyl octanoate (C13, pineapple), suggesting their potential as producers of fruity and chocolate-like aroma compounds.

**Figure 2:**
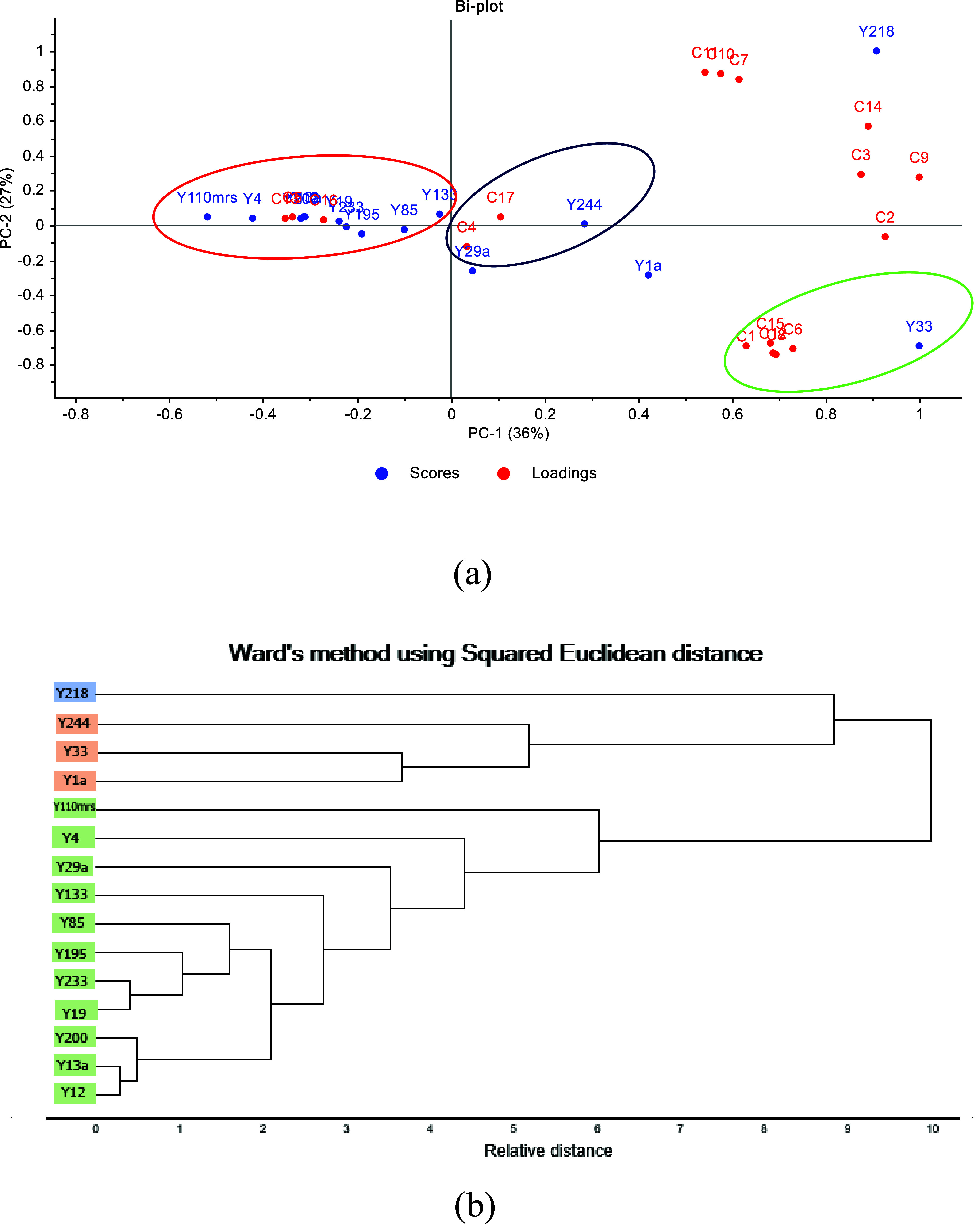
(a) PCA biplot of the volatile compounds produced by yeasts grown in cocoa leachate from Santander, Colombia. The plot shows volatile compounds (red) and yeast strains (blue). (b). Cluster analysis of yeasts based on their VOC production profiles using Ward’s method.

The PCA revealed three distinct clusters: The first (green circle) can be interpreted as an indicator group for selecting yeast strains that produce fruity aromas and ethanol. The metabolic profile of strain Y33 suggests activation of the Ehrlich pathway and the alcohol acetyltransferase route for ester biosynthesis.^35^ The production of compounds such as ethyl dodecanoate, 2-methylpropyl benzoate, 2-phenylethyl acetate, ethyl acetate, and ethanol indicates the active conversion of amino acids and sugars into higher alcohols and esters. In these pathways, amino acids are transaminated and reduced to fusel alcohols, which are then esterified with Acetyl-CoA by alcohol acetyltransferases (Atf1p, Atf2p). The presence of ethyl esters reflects high alcohol acetyltransferase activity, while ethanol formation confirms an efficient glycolytic flux.^36^ Therefore, strain Y33 can be characterized as a high-ester-producing yeast associated with fruity and floral aroma formation. The group associated with floral notes (blue circle) exhibited a metabolic profile consistent with the activation of the Ehrlich pathway and alcohol acetyltransferase reactions responsible for the biosynthesis of higher alcohols and aromatic esters. Compounds such as 3-methyl-2-butanol, ethyl 3-phenyl-2-propenoate, and methyl-2-phenylacetate were derived from amino acid catabolism, particularly valine and phenylalanine, followed by esterification with ethanol or methanol. Meanwhile, 4-methyl-2-phenyl-2-pentenal likely originated from phenylacetaldehyde intermediates through oxidative condensation reactions. These pathways are associated with floral and sweet aroma formation, indicating that this group of strains actively channels amino acid metabolism toward volatile compound synthesis. The third group (red circle), predominantly related to fruity aromas, included strains Y110mrs and Y195, which synthesized 3-methylbutanal via the Ehrlich pathway from leucine degradation, contributing chocolate-like notes.

Cluster analysis (Figure 2b) revealed three distinct clusters. The first cluster comprised strain Y218, notable for producing the highest concentration of 3-phenyl-2-propenoato (10.06 mg/kg) among all yeast strains. Cluster 2 included strains Y33, Y244, and Y1, which contributed to fruity and floral aromas, respectively. Cluster 3, was composed of strains Y4, Y12, Y13a, Y19, Y29a, Y85, Y110mrs, Y133, Y195, Y233, and Y200, all of which were ester producers. This cluster contained the strains Y110mrs and Y195, characterized by their ability to produce 3-methylbutanal and ethyl octanoate, associated with which fruity, particularly pineapple-like, perceptions.

### Changes in the Volatile Profiles of Native Yeasts

When comparing the volatile compounds produced by yeasts in a cocoa leachate (20 g/L) with those reported by Sandoval-Lozano et al. in SDB for the same strains,^10^ differences in both compound concentrations were revealed. A total of 26 volatile compounds were detected in the cocoa leachate medium; however, compounds such as 3-methyl-2-butanol, benzyl acetate, 2-methylpropyl acetate, 2-methylpropyl benzoate, methyl-2-phenylacetate, 3-propenyl 3-phenyl-2-propenoate, 1-phenylethanone, 2,3-butanedione, and 2,3,5,6-tetramethylpyrazine were absent in SDB. Conversely, 36 volatile compounds were identified in the SDB medium, including 3-methyl-1-butanol, 2-phenylethanol, 2,4-di*tert*-butylphenol, 2-pentyl acetate, methylpropyl acetate, ethyl benzoate, diethyl butanedioate, phenylacetaldehyde, 5-methyl-2-phenyl-2-hexenal, 3-hydroxy-2-butanone, 2-heptanone, 2-octanone, 6-methyl-3,5-dihydroxy-2,3-dihydro-4H-pyran-4-one, acetic acid, 3-methylbutanoic acid, 2-methylbutanoic acid, dodecanoic acid, and 2-methylpyrazine, none of which were produced in the cocoa leachate.

After the OAV screening, nine volatile compounds were detected in both culture media. The Mann–Whitney *U* test revealed no significant differences (*p* > 0.05) for ethyl dodecanoate (*p* = 0.061031), ethyl octanoate (*p* = 0.360472), ethyl 3-phenyl-2-propenoate (*p* = 0.852063), 3-methylbutanal (*p* = 0.65475), and 4-methyl-2-phenyl-2-pentenal (*p* = 0.065475). On the other hand, ethanol (*p* = 0.001215), ethyl acetate (*p* = 0.000228), 2-phenylethyl acetate (*p* = 0.000619), and ethyl decanoate (*p* = 0.015099) showed significant differences between media (Figure 3).

**Figure 3:**
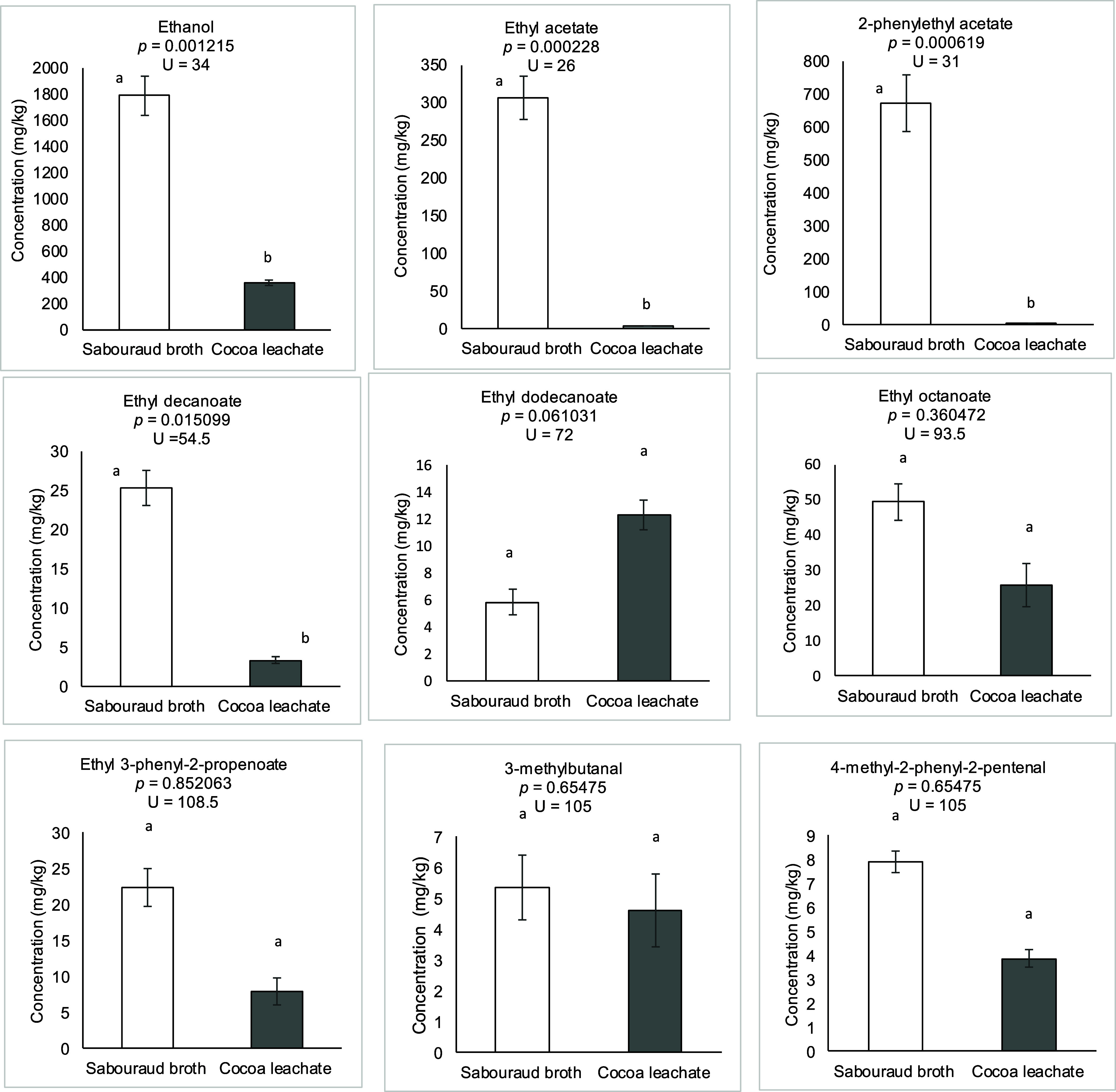
Volatile compound concentrations (mg/kg) produced by yeast in cocoa leachate from the CCN 51 variety and Sabouraud broth. Data are presented as the mean ± standard error (*n* = 15). Different letters indicate significant differences between culture media, *p* ≤ 0.05 by the Mann–Whitney *U* test.

The results show a wide range of concentrations among yeast strains and clear variations between the culture media. While SDB favored the production of ethanol, ethyl acetate, 2-phenylethyl acetate, and ethyl decanoate, cocoa leachate promoted the higher concentration of ethyl dodecanoate (12.344 mg/kg). This suggests that the leachate provides a more favorable environment for its synthesis, possibly due to its diverse sugar composition. Glucose and fructose, the main sugars in cocoa leachate, are metabolized through glycolysis, where they are phosphorylated by hexokinases (HXK1, HXK2) or glucokinase (GLK1), and converted to pyruvate, producing ethanol and CO_2_ under fermentative conditions. Additionally, yeast strains can hydrolyze sucrose into glucose and fructose via β-fructofuranosidase and α-glucosidase, increasing sugar availability for fermentation.^37,38^

The presence of different sugars can influence ester synthesis by providing alternative carbon sources for yeast metabolism.^39^ For example, Scott et al. demonstrated that glucose is preferentially consumed over fructose due to the higher affinity of hexose transporters for glucose, thereby affecting ester production.^40^ Likewise, Moimenta et al. highlighted that ester production is associated with hexose availability and the regulation of nitrogen metabolism during fermentation.^41^ Similarly, Xie et al. reported that different fructose concentrations affect yeast metabolism and the quality of fermented product by shifting carbon flow.^42^

Ethanol production was higher in the SBD medium (1789.83 mg/kg) than in cocoa leachate (355.28 mg/kg), reflecting the faster and more direct metabolism of glucose compared with that of mixed sugars. The presence of multiple sugars in the cocoa leachate (glucose, fructose, and sucrose) likely induced catabolite repression, in which glucose inhibits the metabolism of other carbohydrates, thereby reducing the overall efficiency of volatile compound synthesis.

It has been well established that carbohydrates and amino acid metabolism contribute significantly to VOC production. In this study, the observed differences between SDB and cocoa leachate suggest metabolic modulation. In SDB medium, yeast strains maximized glycolytic flux, resulting in higher ethanol concentrations and consequently greater ethyl acetate synthesis. This short-chain ester is synthesized by the alcohol acetyltransferase (AATase, e.g., ATF1), which directly condenses ethanol with Acetyl-CoA. Conversely, the enhanced formation of long-chain esters, such as ethyl dodecanoate in the cocoa leachate, indicates a redirection of Acetyl-CoA flux. This metabolic shift is attributed to the nutrient and nitrogen limitations inherent to cocoa leachate, which are known to activate the fatty acid synthesis (FAS) pathway for lipid accumulation in yeasts. Under such restrictive conditions, different strains utilize the FAS system to generate the long-chain precursor dodecanoyl-CoA, which is subsequently condensed with ethanol by specific long-chain acyltransferases (e.g., EHT1). Consequently, cocoa leachate acts as a physiological trigger that redirects Acetyl-CoA toward the FAS pathway and promotes the expression of specialized transferases, reflecting a cellular adaptation strategy that directly impacts the final flavor profile by favoring heavy esters over short-chain acetates.

## Conclusions

This study provided useful information for selecting yeasts based on their tendency to produce fruity and chocolate-like aroma compounds, such as Y195 and Y110MRS, or yeasts that generate fruity notes and high ethanol levels. These strains exhibited the best aromatic profiles and are highly suitable candidates for use as starter cultures to enhance the sensory characteristics of cocoa liqueurs and chocolate products.

The use of cocoa leachate proved to be an effective substrate for producing key aroma compounds, such as ethyl octanoate and 3-methylbutanal, which are associated with fruity and chocolate notes. In addition, the absence of long-chain esters such as ethyl dodecanoate in Sabouraud medium highlights the distinctive metabolic capacity of native yeasts cultivated in cocoa leachate. These esters act as aroma fixatives or stabilizers, extending the sensory impact of more volatile compounds and contributing to the greater persistence and aromatic quality in cocoa-derived products.

These findings demonstrate that cocoa leachate is an excellent medium for producing yeast strains, according to their metabolic tendencies. Furthermore, the results obtained using cocoa leachate indicate that this substrate, which serves as the main carbon source under real conditions during cocoa box fermentation, can support the formation of a VOC profile associated with desirable sensory perceptions. Therefore, these findings help reduce uncertainty when screening yeast species for use in field fermentation processes by employing a starter-culture strategy.

## Supplementary Material



## Notes

Screening based on the OAVs of Volatile compounds detected by SPME-GC-MS in yeasts grown in cocoa leachate from CCN 51 (Table S1) (PDF)

## References

[1] El
Jaddaoui I., Rangel D. E. N., Bennett J. W. (2023). Fungal volatiles
have physiological properties. Fungal Biol..

[2] Díaz-Muñoz C., De Vuyst L. (2022). Functional yeast starter
cultures for cocoa fermentation. J. Appl. Microbiol..

[3] El-Ghwas D.
E., Elkhateeb W. A., Akram M., Dada G. M. (2014). Yeast as Biotechnological
Tool in Food Industry. J. Pharm. Res..

[4] Gutiérrez-Ríos H. G., Suárez-Quiroz M. L., Hernández-Estrada Z. J., Castellanos-Onorio O. P., Villegas R. A., Rayas-Duarte P., Sarmiento C. C., Figueroa-Hernández C. Y., Gonzáles-Ríos O. (2022). Yeasts as
Producers of Flavor Precursors during Cocoa Bean Fermentation and
Their Relevance as Starter Cultures: A Review. Fermentation..

[5] Hirko B., Mitiku H., Getu A. (2023). Role of fermentation
and microbes
in cacao fermentation and their impact on cacao quality. Syst. Microbiol. Biomanuf..

[6] Maicas S. (2020). The role of
yeasts in fermentation processes. Microorganisms.

[7] Ordoñez-Araque R. H., Landines-Vera E. F., Urresto-Villegas J. C., Caicedo-Jaramillo C.
F. (2020). Microorganisms
during cocoa fermentation: Systematic review. Foods Raw Mater..

[8] Viesser J. A., de Melo Pereira G. V., de Carvalho Neto D. P., Favero G. R., de Carvalho J. C., Goés-Neto A., Rogez H., Soccol C. R. (2021). Global cocoa fermentation
microbiome: revealing new taxa and microbial functions by next generation
sequencing technologies. World J. Microbiol.
Biotechnol..

[9] Wu Y., Li Z., Zou S., Dong L., Lin X., Chen Y., Zhang S., Chaofan J. I., Liang H. (2023). Chemical Composition
and Flavor Characteristics of Cider Fermented with *Saccharomyces cerevisiae* and Non-*Saccharomyces
cerevisiae*. Foods.

[10] Sandoval-Lozano C. J., Caballero-Torres D., López-Giraldo L. J. (2022). Screening
Wild Yeast
Isolated from Cocoa Bean Fermentation Using Volatile Compounds Profile. Molecules.

[11] Bordet F., Joran A., Klein G., Roullier-Gall C., Alexandre H. (2020). Yeast-yeast interactions: Mechanisms, methodologies
and impact on composition. Microorganisms.

[12] El-Gendi H., Taha T. H., Ray J. B., Saleh A. K. (2022). Recent advances
in bacterial cellulose: a low-cost effective production media, optimization
strategies and applications. Cellulose.

[13] Balladares C., Chóez-Guaranda I., García J., Sosa D., Pérez S., González J. E., Viteri R., Barragán A., Quijano-Avilés M., Manzano P. (2016). Physicochemical characterization of *Theobroma
cacao* L. sweatings in Ecuadorian coast. Emirates J. Food Agric..

[14] Guirlanda C. P., da Silva G. G., Takahashi J. A. (2021). Cocoa honey:
Agro-industrial waste
or underutilized cocoa by-product?. Future Foods.

[15] Saavedra-Sanabria O.
L., Durán D., Cabezas J., Hernández I., Blanco-Tirado C., Combariza M. Y. (2021). Cellulose biosynthesis using simple
sugars available in residual cacao mucilage exudate. Carbohydr. Polym..

[16] Ríos, G. D. ; Saravia, S. S. ; Sandoval-Lozano, C. J. ; López-Girando, L. J. Determinación de las Condiciones para la Producción Vía Fermentativa de Saccharomyces cerevisiae Usando como Fuente de Carbono Lixiviados del Proceso de Fermentación del Cacao; Universidad Industrial de Santander: Santander, Colombia, 2018.

[17] Palencia-Blanco C., Gualdrón-Zambrano A., Guarín-Henao I., Ojeda-Galeano Y., Villamizar-Jaimes A., Zárate-Caicedo D. A. (2020). Proposal for a semi-quantitative method for the determination of
volatile compounds in cocoa liquors. Respuestas.

[18] Akoa S. P., Boulanger R., Onomo P. E., Lebrun M., Ondobo M. L., Lahon M. C. (2023). Sugar profile and volatile aroma composition
in fermented dried beans and roasted nibs from six controlled pollinated
Cameroonian fine-flavor cocoa (*Theobroma cacao* L.) hybrids. Food Biosci..

[19] Escobar S., Santander M., Zuluaga M., Chacón I., Rodríguez J., Vaillant F. (2021). Fine cocoa beans production: Tracking
aroma precursors through a comprehensive analysis of flavor attributes
formation. Food Chem..

[20] Quimbita F., Rodriguez P., Vera E. (2013). Uso del exudado y placenta del cacao
para la obtención de subproductos. Rev.
Tecnol. ESPOL..

[21] Li J., Yuan M., Meng N., Li H., Sun J., Sun B. (2024). Influence of nitrogen status on fermentation performances of non-Saccharomyces
yeasts: a review. Food Sci. Hum. Wellness.

[22] Koné M. K., Guéhi S. T., Durand N., Ban-Koffi L., Berthiot L., Tachon A. F., Brou K., Boulanger R., Montet D. (2016). Contribution of predominant yeasts to the occurrence
of aroma compounds during cocoa bean fermentation. Food Res. Int..

[23] Assi-Clair B. J., Koné M. K., Kouamé K., Lahon M. C., Berthiot L., Durand N., Lebrun S., Julien-Ortiz A., Maraval I., Boulanger R., Guéhi T. (2019). Effect of
aroma potential of *Saccharomyces cerevisiae* fermentation on the volatile profile of raw cocoa and sensory attributes
of chocolate produced thereof. Eur. Food Res.
Technol..

[24] Marseglia A., Musci M., Rinaldi M., Palla G., Caligiani A. (2020). Volatile fingerprint
of unroasted and roasted cocoa beans (*Theobroma cacao* L.) from different geographical origins. Food
Res. Int..

[25] Escobar S., Santander M., Zuluaga M., Chacón I., Rodríguez J., Vaillant F. (2021). Fine cocoa beans production: Tracking
aroma precursors through a comprehensive analysis of flavor attributes
formation. Food Chem..

[26] Wang X. J., Tao Y. S., Wu Y., An R. Y., Yue Z. Y. (2017). Aroma compounds
and characteristics of noble-rot wines of Chardonnay grapes artificially
botrytized in the vineyard. Food Chem..

[27] De
Vuyst L., Leroy F. (2020). Functional role of yeasts, lactic
acid bacteria and acetic acid bacteria in cocoa fermentation processes. FEMS Microbiol. Rev..

[28] Parapouli M., Vasileiadis A., Afendra A. S., Hatziloukas E. (2020). *Saccharomyces cerevisiae* and its industrial applications. AIMS Microbiol..

[29] Utrilla-Vázquez M., Rodríguez-Campos J., Avendaño-Arazate C. H., Gschaedler A., Lugo-Cervantes E. (2020). Analysis of volatile compounds of
five varieties of Maya cocoa during fermentation and drying processes
by Venn diagram and PCA. Food Res. Int..

[30] Zhu H., Wei M., Zhang Y., Tao X. (2024). Analysis of Volatile Organic Compounds
of Different Types of Peppers (*Capsicum annuum* L.) Using Comprehensive Two-Dimensional Gas Chromatography With
Time-of-Flight Mass Spectrometry. eFood.

[31] Solorzano C. Y. E., García D. A. T., Zambrano C. E. E., Moreno-Rojas J. M., Rodríguez Solana R. (2023). Monitoring Changes in the Volatile
Profile of Ecuadorian Cocoa during Different Steps in Traditional
On-Farm Processing. Plants.

[32] Martínez-Avila O., Sánchez A., Font X., Barrena R. (2018). Bioprocesses for 2-phenylethanol
and 2-phenylethyl acetate production: current state and perspectives. Appl. Microbiol. Biotechnol..

[33] Adame-Soto P. J., Aréchiga-Carvajal E. T. M., González-Herrera S. M., Moreno-Jiménez M. R., Rutiaga-Quiñones O. M. (2023). Characterization
of mating type on aroma production and metabolic properties wild *Kluyveromyces marxianus* yeasts. World J. Microbiol. Biotechnol..

[34] Yoshimoto H., Bogaki T. (2023). Mechanisms of production
and control of acetate esters
in yeasts. J. Biosci. Bioeng..

[35] Hazelwood L. A., Daran J. M., Van Maris A. J., Pronk J. T., Dickinson J. R. (2008). The Ehrlich
pathway for fusel alcohol production: A century of research on *Saccharomyces cerevisiae* metabolism. Appl. Environ. Microbiol..

[36] Verstrepen K. J., Van Laere S. D. M., Vanderhaegen B. M. P., Derdelinckx G., Dufour J. P., Pretorius I. S., Winderickx J., Thevelein J. M., Delvaux F. R. (2003). Expression levels of the yeast alcohol
acetyltransferase genes ATF1, Lg-ATF1, and ATF2 control the formation
of a broad range of volatile esters. Appl. Environ.
Microbiol..

[37] Berthels N. J., Otero R. R. C., Bauer F. F., Thevelein J. M., Pretorius I. S. (2004). Discrepancy in glucose and fructose
utilisation during
fermentation by *Saccharomyces cerevisiae* wine yeast strains. FEMS Yeast Res..

[38] Xie D., Lei Y., Sun Y., Li X., Zheng J. (2025). Regulation of fructose
levels on carbon flow and metabolites in yeast during food fermentation. Food Sci. Technol. Int..

[39] Yuan L., Li M., Xu X., Shi X., Chen G., Lao F., Wu J. (2024). Comparative genomics
and fermentation flavor characterization of
five selected lactic acid bacteria provide predictions for flavor
biosynthesis metabolic pathways in fermented muskmelon puree. Food Front..

[40] Scott W. T., Henriques D., Smid E. J., Notebaart R. A., Balsa-Canto E. (2023). Dynamic genome-scale modeling of *Saccharomyces
cerevisiae* unravels mechanisms for ester formation
during alcoholic fermentation. Biotechnol. Bioeng..

[41] Moimenta A.
R., Henriques D., Minebois R., Querol A., Balsa-Canto E. (2023). Modelling
the physiological status of yeast during wine fermentation enables
the prediction of secondary metabolism. Microb.
Biotechnol..

[42] Xie D., Lei Y., Sun Y., Li X., Zheng J. (2025). Regulation of fructose
levels on carbon flow and metabolites in yeast during food fermentation. Food Sci. Technol. Int..

