# Supplementary material for: Production of Volatile Compounds Using Wild Yeasts in a Cocoa Leachate-Based Culture Medium
Source: ACS Omega. 2025 Dec 23;11(1):1348–59. doi: 10.1021/acsomega.5c08827 (PMC12809553; doi:10.1021/acsomega.5c08827)
Supplement: Supplementary file 1 [file ao5c08827_si_001.pdf]

Production of volatile compounds using wild yeasts in a cocoa leachate–based culture medium

Claudia Johanna Sandoval-Lozano[1,2], Yanine Yubisay Trujillo Navarro[2] & Luis Javier Lopez-Giraldo[1*]

[1] Grupo de Investigación en Ciencia y Tecnología de Alimentos, Escuela de Ingeniería Química, Universidad Industrial de Santander, 27th Avenue, 9th Street, Bucaramanga 680006, Santander, Colombia.

[2] Grupo de Investigación en Ingeniería y Tecnología de Alimentos, Facultad de Ingenierías y arquitectura, Universidad de Pamplona, Km 1 University City, Pamplona 543057, Norte de Santander, Colombia.

* Corresponding author: Grupo de Investigación en Ciencia y Tecnología de Alimentos, Escuela de Ingeniería Química, Universidad Industrial de Santander, 27th Avenue, 9th Street, Bucaramanga 680006, Santander. +57(607)6344000 Ext. 3545. Email: ljlopez@uis.edu.co

Suplementary data

Table S1. Screening based on the OAVs of Volatile compounds detected by SPME-GC-MS in yeasts grown in cocoa leachate from CCN 51.

Volatile compunds	OTV	Y1	Y4	Y12	Y13a	Y17	Y19	Y29a	Y33	Y85	Y97	Y110mrs	Y133	Y195	Y200	Y218	233	244
Decanoic acid	1.120	0.168	0.000	10.72	0.000	0.000	0.034	0.462	1.049	0.306	0.021	0.014	0.239	0.000	0.000	0.000	0.029	0.000
2-methylpropanoic acid	2.371	0.284	0.008	0.000	0.032	0.000	0.210	0.653	0.000	0.730	0.000	0.054	0.188	0.000	0.028	0.000	0.394	0.857
Alpha-terpineol	0.330	0.000	0.000	0.161	0.000	0.000	0.000	0.000	0.000	0.000	0.000	0.141	0.000	0.000	0.000	0.000	0.000	0.000
2-pentanol	4.000	0.000	0.000	0.000	0.000	0.000	0.097	0.344	0.000	0.300	0.000	0.045	0.000	0.088	0.000	0.000	0.000	0.000
Ethanol	30.000	0.953	0.046	0.000	0.216	0.104	0.390	1.512	1.865	0.743	0.247	0.211	0.922	0.429	0.143	1.681	0.313	1.991
3-methyl-2-butanol	3.000	1.622	0.070	2.751	0.197	0.000	0.000	0.000	1.633	0.000	0.000	0.016	1.265	0.000	0.174	2.825	0.000	2.762
4-methyl-2-phenyl-2-pentenal	-	n.d	n.d	n.d	n.d	n.d	n.d	n.d	n.d	n.d	n.d	n.d	n.d	n.d	n.d	n.d	n.d	n.d
2-phenyl-2-butenal	1.700	0.123	0.000	0.000	0.000	0.000	0.000	0.164	0.000	0.164	0.022	0.058	0.119	0.000	0.000	0.000	0.000	0.507
Benzaldehyde	0.060	0.000	0.000	0.000	0.000	0.000	0.000	0.000	0.000	0.000	0.000	0.000	0.000	0.000	0.000	0.000	0.000	0.000
3-methylbutanal	0.013	0.000	0.000	0.000	0.000	0.000	0.000	0.000	0.000	0.000	0.000	348.4	0.000	5.267	0.000	0.000	0.000	0.000
Ethyl dodecanoate	1.500	1.400	0.000	0.000	0.000	0.000	0.264	0.932	2.463	0.439	0.000	0.000	0.220	0.574	0.000	0.000	0.329	1.608
Ethyl 3-phenyl-2-propenoate	0.002	348.3	0.000	0.000	0.000	0.000	31.12	0.000	0.000	0.000	0.000	0.000	0.000	0.000	0.000	2850	52.58	0.000
2-methylpropyl benzoate	-	n.d	n.d	n.d	n.d	n.d	n.d	n.d	n.d	n.d	n.d	n.d	n.d	n.d	n.d	n.d	n.d	n.d
3-propenyl 3-phenyl-2-propenoate	-	n.d	n.d	n.d	n.d	n.d	n.d	n.d	n.d	n.d	n.d	n.d	n.d	n.d	n.d	n.d	n.d	n.d
Ethyl 3-phenyl propionate	0.005	0.000	0.000	0.000	30.88	0.000	0.000	0.000	0.000	0.000	0.000	0.000	0.000	0.000	28.25	1596	0.000	0.000
Ethyl decanoate	0.200	0.000	0.000	0.721	0.508	0.000	1.490	20.00	0.000	3.538	0.290	0.000	2.378	0.000	0.283	17.80	0.957	0.000
2-phenylethyl acetate	0.233	4.794	0.000	0.359	0.000	0.000	0.773	1.945	6.933	0.000	0.000	0.000	0.134	1.007	0.000	0.000	0.733	0.000
Ethyl octanoate	0.580	0.000	1.210	0.000	0.000	0.000	0.000	0.000	0.000	0.000	0.000	40.42	0.000	2.813	0.062	0.000	0.000	0.000
Methyl phenylacetate	-	n.d	n.d	n.d	n.d	n.d	n.d	n.d	n.d	n.d	n.d	n.d	n.d	n.d	n.d	n.d	n.d	n.d
Ethyl bencilo	124.000	0.000	0.000	0.001	0.003	0.000	0.005	0.000	0.011	0.000	0.000	0.000	0.000	0.000	0.000	0.000	0.005	0.012
Acetato de etilo	0.940	1.289	0.000	0.000	0.000	0.263	0.000	0.000	1.870	0.000	0.000	0.000	0.000	0.245	0.063	0.000	0.253	0.000
3-methylbutyl acetate	0.160	0.000	4.907	0.000	0.000	0.000	0.000	0.000	0.000	0.000	0.000	0.000	0.000	0.000	0.000	0.000	0.000	0.000
2-methylpropyl acetate	1854.64	0.000	0.000	0.000	0.000	0.000	0.000	0.000	0.003	0.000	0.000	0.000	0.000	0.000	0.000	0.000	0.000	0.000
1-phenylethanone	5.629	0.036	0.000	0.000	0.000	0.000	0.003	0.000	0.000	0.000	0.003	0.000	0.000	0.000	0.006	0.122	0.010	0.044
2.3-butanedione	0.005	0.000	0.000	2.886	2.771	0.000	0.000	0.000	0.000	0.000	0.000	0.000	8.384	0.000	0.000	0.000	0.000	14.62
2.3.5.6-tetramethylpyrazine	38.000	0.001	0.000	0.000	0.000	0.000	0.000	0.001	0.003	0.001	0.000	0.000	0.000	0.000	0.000	0.000	0.000	0.000
2.3.5-trimethylpyrazine	1.800	0.039	0.000	0.000	0.000	0.000	0.000	0.000	0.000	0.000	0.000	0.000	0.000	0.000	0.000	0.000	0.000	0.030

n.d: not detected. Data in bold exceeded the threshold value and contributed to the aromatic profile.
